# Prognostic Value of Routine Hematological Markers and ECOG Performance in Predicting Overall Survival in Lung Cancer—A Retrospective Cohort Study and Literature Review

**DOI:** 10.3390/jcm14217603

**Published:** 2025-10-27

**Authors:** Denisa-Gabriela Ion-Andrei, Alexandra-Cristiana Gache, Elena Mocanu, Andreea-Cristina Postu, Simona-Alina Lupșă, Liliana Mocanu, Cătălina Muntean, Elena Dantes

**Affiliations:** 1 Medical Doctoral School, Faculty of Medicine, Campus—Corp B, Ovidius University of Constanta, 1 University Alley, 900470 Constanta, Romania; denisagabriela94@gmail.com (D.-G.I.-A.); elena.dantes@365.univ-ovidius.ro (E.D.); 2 Department of Pneumology, Faculty of Medicine, Campus—Corp B, Ovidius University of Constanta, 1 University Alley, 900470 Constanta, Romania; 3 Clinical Hospital of Pneumophthysiology, 40 Sentinelei Street, 900002 Constanta, Romaniasimona.lupsa94@gmail.com (S.-A.L.); cata761984@yahoo.com (C.M.); 4 Department of Public Health and Management, Faculty of Medicine, Campus—Corp B, Ovidius University of Constanta, 1 University Alley, 900470 Constanta, Romania; elena.mocanu@univ-ovidius.ro; 5 “St. Apostle Andrew” Emergency County Clinical Hospital, 145 Tomis Street, 900591 Constanta, Romania; lilianamcn@gmail.com

**Keywords:** lung cancer, prognosis, hematological markers, TNM staging, cluster analysis, anemia

## Abstract

**Background**: Accurate prognostic assessment in lung cancer is challenging, especially in settings with limited access to molecular testing. Routine hematological markers may complement TNM staging in identifying high-risk patients. **Methods**: We retrospectively analyzed 304 patients with histologically confirmed lung cancer to evaluate the prognostic value of hematological and inflammatory markers in relation to tumor stage and ECOG performance status. Survival was estimated using Kaplan–Meier analysis and independent predictors were identified through Cox regression. Cluster analysis integrated hematological markers with tumor characteristics. **Results**: Most patients presented with advanced disease (61.2% T4; 57.1% metastatic). Early stages (≤IIC) were associated with nearly double the median overall survival compared with stages ≥IIIA (*p* = 0.001). Nodal involvement and metastases further stratified outcomes. Among hematological markers, anemia (Hb < 11.85 g/dL) and leukocytosis (WBC > 11.71 × 10^9^/L) predicted shorter survival and remained independent predictors in the multivariable Cox model (HR 1.70, *p* < 0.001; HR 1.54, *p* = 0.004), along with T4 stage (HR 1.47, *p* = 0.015). PLT count and fibrinogen were significant in univariate analysis but not after adjustment, while CRP and D-dimer showed no association. Cluster analysis identified two subgroups, with patients in Cluster 2 (T4 stage, anemia, leukocytosis) experiencing more than double the mortality risk compared with Cluster 1 (HR 2.33, *p* < 0.001). **Conclusions**: TNM stage remains the dominant prognostic factor in lung cancer. However, Hb and WBC count provide additional prognostic information, and cluster analysis may refine risk stratification by capturing clinically relevant heterogeneity.

## 1. Introduction

Lung cancer remains a major global health challenge, ranking as the second most commonly diagnosed malignancy and the leading cause of cancer-related mortality. In 2020 alone, it accounted for an estimated 2.2 million newly diagnosed cases and 1.8 million deaths worldwide, underscoring its substantial contribution to the global burden of disease [1,2]. Both incidence and mortality vary markedly across geographical regions and between sexes, reflecting differences in exposure to risk factors, healthcare access and early detection practices.

In Eastern Europe, lung cancer outcomes are particularly poor due to high smoking prevalence, delayed diagnosis, and limited access to molecular or imaging-based testing. Consequently, most patients are diagnosed at advanced stages, and survival remains significantly lower than in Western Europe. These disparities highlight the need for simple, accessible, and reproducible prognostic tools that could support individualized management even in resource-limited healthcare systems [3].

Routine laboratory parameters—such as hemoglobin (Hb), white blood cell (WBC) and platelet counts (PLT), C-reactive protein (CRP), fibrinogen and D-dimer—offer indirect but clinically relevant evidence of these mechanisms and are available in everyday practice [4,5,6]. Low Hb and albumin levels are established indicators of cancer-associated cachexia, while elevated CRP, fibrinogen, and D-dimer mirror inflammatory and thrombotic activity within the tumor microenvironment. Their ubiquity and low cost make them attractive candidates for pragmatic prognostic modeling, particularly in low- and middle-income contexts [7].

Functional performance, most commonly assessed using the Eastern Cooperative Oncology Group (ECOG) score, remains a cornerstone in survival prediction for lung cancer patients. Poor performance status (ECOG ≥ 2) is associated with a markedly reduced ability to tolerate aggressive systemic therapy, with median survival rarely exceeding 6–12 months in large observational cohorts [8,9].

However, current prognostic models often depend on advanced molecular profiling or imaging biomarkers which are not routinely available in many healthcare systems. Few studies have systematically evaluated combinations of accessible laboratory parameters and clinical indicators within a unified prognostic framework, and even fewer have done so in Eastern European populations, where late diagnosis and restricted access to novel therapies remain prevalent [10,11,12,13].

The present study aimed to evaluate the prognostic significance of four routinely measured hematological parameters—Hb, CRP, fibrinogen and D-dimer—together with ECOG performance status in patients with lung cancer treated at a regional oncology center in Eastern Europe. Although prior evidence regarding CRP and D-dimer has been inconsistent, both markers were included due to their biological plausibility and clinical availability. Their assessment alongside TNM staging was intended to determine their potential additive value in refining prognostic accuracy. By integrating these inexpensive, reproducible variables into a composite prognostic model, we sought to provide a pragmatic and regionally adaptable framework for early mortality risk stratification in lung cancer.

An internal validation process was undertaken to assess the predictive accuracy of the model, with the ultimate goal of supporting timely, evidence-based therapeutic decision-making, particularly in healthcare settings with limited access to advanced diagnostic resources.

## 2. Materials and Methods

### 2.1. Study Design and Setting

This retrospective, observational, single-center cohort study was conducted at the Constanta Clinical Hospital for Pneumophthisiology in Romania, a regional referral center for respiratory diseases. This study included patients diagnosed and/or treated between January 2023 and June 2025. All data were collected and analyzed retrospectively from existing medical records, with a predefined cut-off date of 1 June 2025. Consequently, survival analysis was performed on complete, verified data available up to that date, ensuring that no prospective follow-up or ongoing cases were included.

### 2.2. Study Population

From an initial pool of 602 patients with histologically confirmed lung cancer (including both non-small cell lung cancer [NSCLC] and small cell lung cancer [SCLC]), only those with complete clinical, laboratory and follow-up data—including a documented date of death—were eligible for inclusion in the survival analysis. The final study cohort comprised 304 individuals.

Patients were considered eligible for the present study if they fulfilled all of the following conditions:

1. Histological confirmation of primary lung cancer, either NSCLC or SCLC, as defined by the latest World Health Organization (WHO) classification of thoracic tumors.
2. Adult age group, defined as 18 years or older at the time of initial diagnosis.
3. Comprehensive and verifiable clinical documentation, including complete demographic data, medical history, oncological staging, and follow-up information, with explicit confirmation of survival status at the last contact.
4. Baseline hematological evaluation performed within 14 days after histological diagnosis, encompassing all parameters of interest for this study (Hb, WBC, PLT count, CRP, fibrinogen, erythrocyte sedimentation rate—ESR, 30/60 min, and D-dimer).

These criteria were established to ensure the accuracy, consistency and comparability of data across the study cohort, thereby strengthening the internal validity of the survival analyses.

Patients were excluded from this study if they met any of the following conditions:

1. Presence of secondary lung malignancies, defined as pulmonary metastases originating from extrapulmonary primary tumors.
2. Incomplete or insufficient clinical documentation, including missing key variables such as survival status, baseline hematological parameters, or ECOG performance score.
3. Concurrent medical conditions with the potential to confound laboratory results, such as active hematological disorders, ongoing acute infections, or chronic inflammatory diseases unrelated to lung cancer.
4. History of other active malignancies diagnosed within the previous five years, except non-melanoma skin cancers or in situ cervical carcinoma, to avoid the influence of competing cancer-related mortality.
5. Baseline hematological evaluation was performed outside the predefined time window of 14 days from histological diagnosis, ensuring temporal consistency between diagnostic confirmation and laboratory assessment.

These exclusion criteria were applied to reduce confounding factors, enhance the reliability of prognostic evaluations, and maintain methodological rigor throughout this study.

### 2.3. Data Collection and Variables

Demographic, clinical and laboratory data were retrieved from the hospital’s electronic medical records and oncology registry. The following variables were analyzed:

- Hematological parameters: Hb, WBC count, PLT count, CRP, ESR, 30/60 min, fibrinogen, and D-dimer. Although serum albumin is a well-established prognostic biomarker in lung cancer, it was not included in the multivariable analysis because baseline albumin levels were missing for approximately one-third of the cohort. To minimize data loss and potential selection bias, only hematological parameters with complete baseline values were retained.
- Functional status: ECOG performance status (scale 0–5).
- Comorbidities: chronic obstructive pulmonary disease (COPD), cardiovascular disease, diabetes mellitus, and other significant chronic illnesses.
- Outcome: Overall survival (OS), defined as the time from histological diagnosis to death from any cause or last follow-up (censored for patients alive at the study endpoint).

### 2.4. Statistical Analysis

Data analysis was performed using IBM SPSS Statistics version 25, with figures generated in Microsoft Office Excel and Word 2024. Categorical variables were expressed as counts and percentages and compared using Fisher’s exact test. Continuous variables were expressed as medians with interquartile ranges (IQR) or means with standard deviations, depending on distribution; normality was assessed using the Shapiro–Wilk test. Between-group comparisons for non-normally distributed variables were performed using the Mann–Whitney U test.

Receiver operating characteristic (ROC) curves were constructed for significant continuous predictors, with the area under the curve (AUC) and 95% confidence intervals (CI) calculated. Cut-off values were determined using Youden’s index, with corresponding sensitivity and specificity reported. Kaplan–Meier survival curves were plotted, and differences between groups were assessed using the log-rank test.

Cox proportional hazards regression analysis was applied to identify independent predictors of mortality. Variables significant in univariable analysis (*p* < 0.05) were entered into a multivariable model using forward stepwise selection (Wald method).

Multivariable Cox proportional hazards models were constructed using a stepwise forward selection procedure, retaining only variables with *p* < 0.05 and variance inflation factor (VIF) < 2 to minimize multicollinearity and overfitting. Cluster analysis was performed as an exploratory technique to identify homogeneous subgroups within the cohort. Hierarchical clustering was first applied using Ward’s method to estimate the optimal number of clusters based on the dendrogram structure and silhouette coefficients. K-means clustering was subsequently used to refine these groupings using continuous hematological and clinical variables. Latent class analysis was not adopted because it relies on categorical variables and requires larger sample sizes for model stability.

A two-tailed *p*-value < 0.05 was considered statistically significant for all analyses.

### 2.5. Ethics Statement

This study was approved by the Institutional Ethics Committee of the Constanta Clinical Hospital for Pneumophthisiology (Approval No. 6744/02.07.2024) and was conducted in accordance with the Declaration of Helsinki. Informed consent was obtained from all participants for the use of anonymized medical data for research purposes.

## 3. Results

### 3.1. Patient Characteristics

A total of 304 patients with histologically confirmed lung cancer were included in this study. According to the table, 68.4% of the patients were men, with a median age of 67 years (IQR = 62–72), living in an urban environment (59.9%). Most of the patients were active smokers (52%) or former smokers (31.2%), with a median pack-years value of 31 (IQR = 20–45). 36.8% of the patients had job exposure. ECOG performance status at diagnosis ranged from 0 to 3, with a median score of 1.

Detailed demographic and baseline clinical characteristics are provided in Table 1.

### 3.2. Tumor Location

In terms of anatomical distribution, tumors were most frequently located in the right lung (54.6%), particularly in the upper lobe (33.2%). The left lung accounted for 45.4% of cases, with the upper lobe also being the predominant site (27.6%). Bilateral involvement was rare. The detailed distribution of tumor sites is shown in Table 2.

### 3.3. Radiological Appearance

Radiological assessment revealed that the most common presentation was a pulmonary mass (64.5%). Patients who had pulmonary nodes were more frequently associated with survival (16.5% vs. 6.1%, *p* = 0.022). These imaging characteristics are summarized in Table 3.

### 3.4. TNM Classification and Survival

With respect to tumor staging, most patients presented with advanced disease, with T4 tumors in 61.2% of cases and distant metastases in 57.1%. Early-stage disease was less common but conferred a clear survival advantage; for example, stage IB accounted for 6.6% of survivors compared with only 0.9% among deceased patients, whereas stage IVC was strongly associated with mortality (9.9% vs. 2.2%, *p* = 0.005). The complete stage-specific distribution of survivors and deceased patients according to the TNM classification is provided in Supplementary Table S1.

Nodal involvement further stratified outcomes: patients with N0 disease showed significantly better survival (37.4% vs. 18.0%, *p* = 0.006), while N2 disease was the most frequent nodal stage (44.4%). Metastatic status also had a major prognostic impact. Patients without metastases were more likely to survive (50.6% vs. 37.3%, *p* = 0.010), whereas combined pulmonary and extrapulmonary metastases were strongly associated with death (26.9% vs. 12.4%).

**Figure 1:**
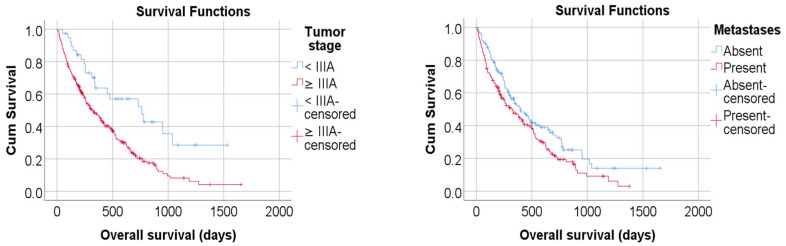
Kaplan–Meier Curves by TNM Stage and metastasis. Kaplan–Meier survival curves illustrating overall survival according to TNM stage. Each curve represents a distinct TNM group (<IIIA—IA/IB/IIA/IIB vs. ≥IIIA—IIIA/IIIB/IIIC/IVA/IVB/IVC). The *x*-axis shows time in months from histological diagnosis, and the *y*-axis indicates cumulative survival probability. Censored observations are marked with vertical ticks. Survival distributions were compared using the log-rank test (*p* < 0.001).

Kaplan–Meier analysis confirmed these associations, showing that patients with stage ≤IIC had nearly double the median overall survival compared with those at stage ≥IIIA (*p* = 0.001). Survival curves according to TNM stage and metastatic status are illustrated in Figure 1 and detailed distributions are provided in Table 4. Detailed Kaplan–Meier survival statistics for tumor aspect, T4 stage, nodal and metastatic status are presented in Supplementary Table S2.

### 3.5. Hematological and Inflammatory Parameters in Survivors vs. Deceased Patients

Median values of hematological and inflammatory markers are presented in Table 5. Graphical comparisons of hemoglobin, leukocyte, platelet and fibrinogen levels according to survival status are illustrated in Supplementary Figure S1.

Compared with survivors, deceased patients exhibited significantly lower Hb levels and mean corpuscular volume, as well as higher WBC and PLT counts and fibrinogen concentrations (all *p* < 0.05). MCHC also showed a slight but statistically significant increase in deceased patients. In contrast, ESR, CRP, and D-dimer did not differ significantly between groups.

These findings indicate that anemia, leukocytosis, thrombocytosis and elevated fibrinogen levels are more strongly associated with poor prognosis, whereas inflammatory and coagulation markers such as CRP and D-dimer have limited discriminative value in this cohort. Corresponding ROC curve analyses for mortality prediction using hematological parameters are summarized in Supplementary Table S3.

**Figure 2:**
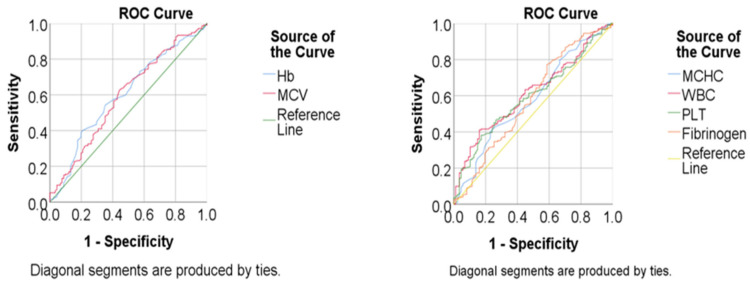
ROC Curves for mortality prediction (hematological parameters). Receiver Operating Characteristic (ROC) curves illustrating the predictive performance of baseline hematological markers for mortality in lung cancer patients. ROC curves for hemoglobin (Hb) and mean corpuscular volume (MCV). ROC curves for mean corpuscular hemoglobin concentration (MCHC), white blood cell count (WBC), platelet count (PLT), and fibrinogen. The *x*-axis represents 1-specificity, and the *y*-axis represents sensitivity.

Kaplan–Meier survival analysis demonstrated that anemia (Hb < 11.85 g/dL) and leukocytosis (WBC > 11.71 × 10^9^/L) were strongly associated with shorter overall survival (both *p* < 0.001). Thrombocytosis (PLT > 358.5 × 10^9^/L) and hyperfibrinogenemia (>4.06 g/L) were also significantly correlated with poorer outcomes (*p* = 0.021 and *p* = 0.032, respectively). By contrast, CRP and D-dimer did not show significant associations with survival. Representative survival results and curves for Hb and WBC count are presented in Table 6 and Figure 2.

### 3.6. Cox Regression Analysis

In the univariate Cox regression analysis, several parameters were associated with increased mortality risk, including advanced tumor stage, nodal involvement, presence of metastases, anemia, leukocytosis, thrombocytosis and elevated fibrinogen (all *p* < 0.05). However, in the multivariable model, only three factors remained independent predictors of mortality: T4 tumor stage (HR 1.47, 95% C.I.: 1.08–2.01, *p* = 0.015), anemia defined as Hb < 11.85 g/dL (HR 1.70, 95% C.I.: 1.27–2.27, *p* < 0.001), and leukocytosis defined as WBC > 11.71 × 10^9^/L (HR 1.54, 95% C.I.: 1.15–2.05, *p* = 0.004).

These results emphasize that, beyond TNM staging, routine hematological markers such as Hb and WBC count provide independent prognostic information in lung cancer. The detailed results of the univariate and multivariable analyses are shown in Table 7.

### 3.7. Cluster Analysis

**Figure 3:**
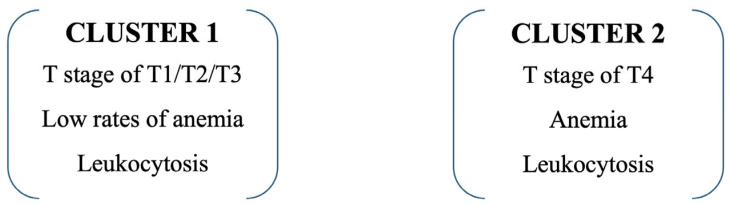
Cluster Analysis of Hematological Profiles. Unsupervised cluster analysis of hematological and clinical parameters identifying distinct patient subgroups. Cluster 1 included patients with low rates of anemia and leukocytosis, T1–T3 stages, while Cluster 2 comprised those with T4, anemia and leukocytosis.

Cluster analysis integrating tumor stage and hematological parameters identified two distinct patient subgroups with significantly different clinical profiles. Cluster 1 included predominantly patients with less advanced disease, while Cluster 2 was defined by a high prevalence of T4 tumors (87.1% vs. 40.9%, *p* < 0.001), anemia (58.3% vs. 15.8%, *p* < 0.001), and leukocytosis (80.6% vs. 11.7%, *p* < 0.001). Patients assigned to Cluster 2 exhibited a more aggressive disease pattern and were associated with significantly poorer survival outcomes compared with Cluster 1. Detailed cluster characteristics and distances between final cluster centers are provided in Supplementary Table S4. The clinical distribution of these defining variables is presented in Table 8, while the graphical representation of cluster assignment is shown in Figure 3.

Using a univariable Cox-proportional hazard regression model, with type of cluster as an independent variable, shows that patients in Cluster 2 have an increased risk of death by 2.334 times (95% C.I. = 1.764–3.089) than patients in Cluster 1.

## 4. Discussions

### 4.1. Summary of Main Findings

In this retrospective cohort of 304 patients with lung cancer, we evaluated the prognostic role of routine hematological markers in the context of TNM classification and ECOG performance status. Our main findings can be summarized as follows. First, tumor stage remained the strongest determinant of survival, with patients diagnosed at stage ≤IIC having nearly double the median overall survival compared with those at stage ≥IIIA. Nodal involvement and the presence of distant metastases were likewise associated with significantly poorer outcomes, confirming the well-established prognostic gradient across TNM categories.

Second, among the hematological parameters, anemia (Hb < 11.85 g/dL) and leukocytosis (WBC > 11.71 × 10^9^/L) were identified as independent predictors of mortality in the multivariable Cox model, together with T4 tumor stage. PLT count and fibrinogen showed prognostic value in univariate and Kaplan–Meier analyses but did not retain significance after adjustment for other variables, while CRP and D-dimer were not significantly associated with survival.

Third, the application of cluster analysis integrating tumor stage with hematological parameters revealed two distinct subgroups with markedly different prognoses. Patients in Cluster 2, characterized by advanced disease (T4), anemia, and leukocytosis, had more than a two-fold higher risk of death compared with those in Cluster 1.

Taken together, these results emphasize that, beyond conventional TNM staging, simple and routinely available hematological markers can refine prognostic assessment. The integration of such variables through cluster analysis may help capture clinically relevant heterogeneity and provide a pragmatic framework for individualized risk stratification in lung cancer.

Although several hematological markers showed significant associations with survival in univariate analyses, only three retained independent prognostic value in the multivariable Cox model. This likely reflects the moderate sample size and the interdependence between inflammatory and tumor-related variables. The stepwise approach was applied to ensure model robustness and to prevent overfitting.

### 4.2. Comparison with Previous Literature

Numerous studies have confirmed the prognostic significance of D-dimer, fibrinogen and CRP in lung cancer, with abnormal levels consistently associated with reduced overall survival [8,14,15].

#### 4.2.1. Survival According to TNM Stages

The study results revealed marked differences in survival according to tumor stage. Patients diagnosed at early stages (IA, IB, IIA, IIB) had a higher probability of survival, being more frequently alive at the time of analysis (e.g., survivors 6.6% in IB vs. 0.9% among deceased). In contrast, advanced stages, particularly IVC (9.9% vs. 2.2%, *p* = 0.005), were strongly associated with higher mortality. Kaplan–Meier analysis confirmed that median survival for patients in stages ≤IIC was nearly double compared with those in ≥IIIA, emphasizing the importance of early detection. These findings align with population-based data, such as SEER and European registries, which show that 5-year survival exceeds 60% for patients diagnosed at stage I but drops below 10% for those at stage IV [16].

Our findings regarding the poor survival outcomes of patients with stage IV disease are consistent with the recent analysis by Ayoade et al. (2025), which documented meaningful survival gains in stage IV NSCLC across sociodemographic strata. Using population-based data, the authors demonstrated that although stage IV remains associated with dismal overall survival, advances in systemic therapy—particularly immunotherapy and targeted approaches—have led to incremental improvements in survival over the past decade. Importantly, these benefits were not equally distributed, with disparities observed by age, race and socioeconomic status, highlighting the persistent inequities in access to novel therapies [17]. This supports the idea that prognostic models based on easily accessible biomarkers, like those analyzed in our study, hold significant value in environments where molecular testing and expensive therapies are not readily available.

#### 4.2.2. Nodal Status and Metastases

Regarding lymph node involvement, patients with N0 had significantly better survival (37.4% vs. 18%, *p* = 0.006) compared to those with N2/N3 disease, where the risk of death was markedly increased. The presence of metastases was a significant negative prognostic factor: patients without metastases had a survival rate of 50.6% compared to 37.3% in those with dissemination, while combined pulmonary and extrapulmonary metastases were associated with the poorest outcomes (26.9% vs. 12.4%, *p* = 0.010).

This distribution is consistent with findings from multicenter European and Asian studies, where metastatic status is considered the strongest predictor of overall survival [18].

#### 4.2.3. Cluster Analysis

The application of cluster analysis in our cohort enabled the stratification of patients into two distinct prognostic subgroups. Cluster 1 was characterized by less advanced stages (T1–T3), absence of anemia, and absence of leukocytosis and was associated with a more favorable prognosis. In contrast, Cluster 2 consisted predominantly of patients with T4 tumors, frequently presenting anemia and leukocytosis, and demonstrated a 2.3-fold higher risk of death (HR = 2.334, 95% CI: 1.764–3.089). Comparable findings have been reported in external cohorts. Wang et al. (2023) showed that the integration of hematological and inflammatory markers into cluster analysis identified subgroups with distinct therapeutic responses and survival trajectories in SCLC [19]. Similarly, a real-world analysis published by Li et al. (2024) confirmed that combining clinical and biological data through clustering allows more refined risk stratification in extensive-stage SCLC, identifying cohorts with median survival differences of up to 8 months [20].

These findings highlight the added prognostic value of unsupervised learning techniques such as clustering, which can detect clinically relevant heterogeneity beyond TNM staging. By integrating simple laboratory markers with tumor characteristics, cluster-based approaches provide a pragmatic framework for risk-adapted treatment strategies and patient counseling in lung cancer.

#### 4.2.4. D-Dimer

D-dimer represents a fragment produced during the dissolution of blood clots, reflecting ongoing activation of both coagulation and fibrinolytic pathways [21].

Prospective and retrospective studies consistently support its prognostic relevance. Ay et al. (2012), in the extensive Cancer and Thrombosis Study (CATS), including 1178 patients with various malignancies, demonstrated that elevated baseline D-dimer levels independently predicted reduced overall survival, with a hazard ratio (HR) of approximately 1.5 per doubling of the value [8].

A meta-analysis by Ma et al. (2021), encompassing 28 studies with a total of 8452 lung cancer patients, demonstrated that elevated plasma D-dimer levels were significantly associated with reduced overall survival (HR = 1.742, 95% CI: 1.542–1.969, *p* < 0.001) and progression-free survival (HR = 1.385, 95% CI: 1.169–1.641, *p* = 0.003) compared with lower levels. Subgroup analyses indicated that tumor localization, detection methodology, and disease stage influenced the strength of this association. These results provide robust evidence supporting high plasma D-dimer as an independent negative prognostic biomarker in lung cancer [22].

In surgical cohorts, Li et al. (2022) found that lower preoperative D-dimer levels were associated with significantly longer survival in stage I–II NSCLC after complete resection, indicating prognostic relevance even in early-stage disease. In a separate retrospective study of 277 patients with advanced NSCLC, the same group stratified patients by pretreatment plasma D-dimer levels—normal (≤0.5 µg/mL) versus elevated (>0.5 µg/mL)—and demonstrated that high D-dimer was independently associated with shorter progression-free survival (PFS) and overall survival (OS) (*p* < 0.01). After propensity score matching, elevated D-dimer remained strongly linked to poorer outcomes, with median PFS of 6.4 versus 11.5 months (HR = 1.70, 95% CI: 1.25–2.37, *p* < 0.001) and median OS of 12.7 versus 30.4 months (HR = 2.29, 95% CI: 1.54–3.41, *p* < 0.001). Collectively, these findings underscore the prognostic utility of D-dimer across both early- and advanced-stage NSCLC [23].

However, in our cohort, D-dimer did not remain an independent prognostic significance after multivariable adjustment, suggesting that its apparent effect may be confounded by tumor stage and overall biological status. While D-dimer reflects cancer-related hypercoagulability, its effectiveness as an independent prognostic biomarker should be interpreted with caution and needs further validation through larger, multicenter studies.

The discrepancy between our results and those of previous large-scale studies may reflect methodological and cohort-specific differences. Unlike meta-analyses that included heterogeneous populations and early-stage disease, our cohort predominantly consisted of patients with advanced or metastatic tumors, in whom D-dimer elevation is almost universal and therefore less discriminative. In addition, differences in assay standardization and cutoff definitions may have reduced the statistical sensitivity to detect subtle prognostic gradients.

#### 4.2.5. Fibrinogen

Fibrinogen has been widely investigated as a prognostic biomarker in lung cancer, with consistent evidence linking elevated levels to adverse survival outcomes. Zhong et al. (2018), in a meta-analysis of 16 studies including 6881 patients, reported that high plasma fibrinogen was significantly associated with worse overall survival (HR = 1.38, 95% CI: 1.22–1.55, *p* < 0.001) and shorter disease-free or progression-free survival (HR = 1.29, 95% CI: 1.01–1.65, *p* = 0.042). The association persisted in the NSCLC subgroup, where elevated fibrinogen predicted poorer OS (HR = 1.32, 95% CI: 1.14–1.53, *p* < 0.001) [15]. Similarly, Zhang et al. (2019) analyzed 20 studies involving 6494 patients and confirmed the adverse prognostic impact of high fibrinogen, with pooled HRs of 1.44 for OS, 1.49 for PFS, and 1.69 for DFS. Moreover, elevated fibrinogen correlated with aggressive tumor features, including lymph node metastasis and advanced stage [24].

The prognostic value of fibrinogen has also been demonstrated in specific clinical contexts. Sinn et al. (2022) evaluated patients with stage III/N2 NSCLC treated with neoadjuvant therapy and found that post-treatment fibrinogen > 400 mg/dL was associated with significantly shorter OS (28.2 vs. 60.9 months; HR ≈ 1.78, *p* = 0.048). Interestingly, a decline in fibrinogen following therapy independently predicted better survival, suggesting that changes in fibrinogen may reflect treatment response [25]. In early-stage disease, Mitsui et al. (2022) reported that high preoperative fibrinogen was linked to worse survival even after complete resection and was associated with pathological features of tumor aggressiveness, such as lymphovascular invasion [26].

Our results align with this body of evidence, showing that elevated fibrinogen levels were associated with early mortality in lung cancer patients, though not independently in multivariable analysis. Taken together, these findings highlight fibrinogen’s role not only as a marker of systemic inflammation and hypercoagulability but also as a surrogate for underlying tumor biology. Its routine availability and low cost further support its consideration in prognostic models, particularly in settings where access to molecular or advanced imaging biomarkers is limited.

#### 4.2.6. CRP

Multiple studies have established a strong association between elevated CRP levels and poor survival outcomes in lung cancer.

In a bi-center retrospective study of patients with NSCLC treated with PD-1/PD-L1 inhibitors, Riedl et al. (2020) reported that elevated baseline CRP was an independent predictor of poor prognosis, being significantly associated with reduced progression-free survival (PFS) and overall survival (OS), with hazard ratios per doubling of CRP of 1.37 for PFS and 1.42 for OS [27].

Regarding SCLC, Stensvold et al. (2021) analyzed a Danish registry including 923 patients. They found that pretreatment CRP levels above the clinical threshold (>8 mg/L) were significantly associated with worse overall survival (adjusted HR = 1.25, 95% CI: 1.08–1.46) [14].

More recent evidence, such as that provided by Onodera et al. (2023), suggests that elevated CRP levels prior to initiation of immune checkpoint inhibitor (ICI) therapy are linked not only to poorer prognosis but also to a higher incidence of immune-related adverse events (irAEs). High CRP was significantly associated with shorter survival, with median PFS of 2.2 versus 3.3 months and median OS of 8.9 versus 39.1 months [28].

Our findings are in partial agreement with these observations, as elevated CRP (>20 mg/L) was associated with early mortality in our cohort. This association likely reflects the impact of a systemic pro-tumorigenic inflammatory milieu, which contributes to angiogenesis, immune evasion and metastatic spread. However, in contrast to the consistent results reported in the literature, CRP did not remain an independent predictor in our multivariable model. This indicates that its clinical utility should be assessed alongside other parameters such as Hb, WBC and tumor T4, which more accurately capture the independent prognostic signal in our cohort.

The lack of independent prognostic value of CRP in our multivariable model contrasts with several studies reporting its significance. This inconsistency may be explained by variations in patient selection, disease stage, and cutoff thresholds across studies. As most individuals in our cohort presented with late-stage disease, the uniformly elevated inflammatory status might have diminished the discriminatory power of CRP. Furthermore, the moderate sample size could have limited our ability to detect weaker associations.

#### 4.2.7. Integration of Hematological Biomarkers with ECOG Performance Status

Only a limited number of studies have explicitly combined inflammatory or coagulation-related biomarkers with ECOG performance status into a single prognostic framework.

A relevant example is provided by a 2023 retrospective study of patients with SCLC treated with etoposide–cisplatin–based chemotherapy. The authors assessed risk factors for febrile neutropenia (FN) and identified ECOG performance status, together with nutritional status and inflammatory markers, as independent predictors of FN risk (*p* < 0.001 for the overall model; *p* = 0.019 for ECOG-PS). This integrative approach illustrates the value of combining biological, clinical, and functional parameters for patient risk stratification [29].

Similarly, in our study, the incorporation of ECOG performance status with routine hematological biomarkers provided a comprehensive prognostic framework, capturing both tumor-related biological activity and patient functional reserve.

### 4.3. Limitations

This study has several important limitations. First, its retrospective and single-center design limits the generalizability of the findings, as the patient population and management strategies may not fully reflect those in other institutions or regions. This design allowed comprehensive data collection and consistent laboratory testing. Future multicenter prospective studies are required to validate the reproducibility and external applicability of these findings.

Another important limitation is the lack of external validation. Although internal validation was performed through ROC, Kaplan–Meier, and multivariable Cox analyses, the predictive performance of the proposed model requires confirmation in independent, multicenter cohorts. Future studies with larger and more diverse populations are needed to verify its generalizability and clinical utility.

Furthermore, the analysis included only complete cases, which may have introduced selection bias and potentially led to unrepresentative estimates of prognostic associations. Survival analysis was restricted to patients for whom complete mortality data, including the exact date of death, were available. As a result, only 304 of the 602 eligible cases were included in the survival assessment, which may further limit applicability to the broader lung cancer population. The exclusion of nearly half of the screened population—mainly due to missing laboratory or follow-up information—may have introduced a selection bias favoring cases with more comprehensive clinical documentation. To mitigate this concern, baseline characteristics such as age, sex, and histological subtype were compared between included and excluded patients, revealing no substantial differences. Nevertheless, this limitation should be acknowledged when generalizing the study findings to the broader lung cancer population.

Treatment-related variables, including surgery, chemotherapy, and immunotherapy, were not included in the analysis because these data were incomplete in the registry and outside the primary focus of this study. Our objective was to assess the prognostic impact of routinely available hematological and clinical parameters at diagnosis, independent of subsequent therapeutic interventions. Nevertheless, we acknowledge that treatment heterogeneity may have influenced survival outcomes, and future prospective studies should address this aspect.

Another limitation is the absence of imaging-based or molecular biomarkers, such as radiomics features or genomic alterations, which have been shown to enhance the accuracy of prognostic models in lung cancer. The inclusion of such parameters, along with external validation in independent, multicenter cohorts, would strengthen the robustness and clinical applicability of the proposed model. Future research should aim to integrate routine hematological parameters with advanced molecular and imaging biomarkers to develop comprehensive, multimodal prognostic tools capable of informing individualized treatment strategies.

### 4.4. Clinical Implications and Future Directions

The prognostic model developed in this study, integrating four routinely measured hematological biomarkers with ECOG performance status, offers a pragmatic and cost-effective tool for survival risk stratification in lung cancer. All included variables are inexpensive, widely available and part of standard clinical workflows, which makes the model particularly relevant in resource-limited healthcare systems where access to molecular testing, advanced imaging, or targeted therapies is restricted.

By combining objective laboratory measurements with functional status, the model captures both tumor-related biological activity and the patient’s physiological reserve—two complementary dimensions that critically influence treatment tolerance and survival outcomes. In clinical settings, such an approach could support early identification of high-risk patients, guiding treatment decisions toward tailored strategies, such as prioritizing supportive care, closer follow-up or targeted interventions to improve functional capacity.

Furthermore, the simplicity of the model facilitates its integration into routine oncology practice without the need for specialized equipment or expertise, enabling its potential adoption in both tertiary cancer centers and peripheral hospitals. With external validation in diverse populations, this approach could contribute to more equitable prognostic assessment and better allocation of healthcare resources in lung cancer management.

The integration of routine hematological markers into existing prognostic systems such as TNM staging and ECOG performance status may enhance their predictive accuracy. While TNM classification provides an anatomical assessment of disease burden, parameters such as Hb and WBC count reflect the patient’s systemic and inflammatory status, offering additional biological context. Incorporating these readily available variables could help identify high-risk patients within the same TNM stage, guiding follow-up intensity and treatment decisions. This combined approach may be particularly valuable in healthcare settings with limited access to molecular or imaging biomarkers.

## 5. Conclusions

In this retrospective analysis, we evaluated the prognostic value of routine hematological markers (Hb, CRP, fibrinogen, D-dimer) integrated with ECOG performance status in patients with lung cancer. The results demonstrated that anemia (Hb < 11.85 g/dL) and leukocytosis (WBC > 11.71 × 10^9^/L), together with the presence of tumor T4, were independent predictors of mortality. CRP, fibrinogen and D-dimer showed significant associations in univariate and Kaplan–Meier analyses, but not in the multivariate model, suggesting that other clinico-biological factors influence their prognostic value.

Integrating hematological markers with ECOG performance status remains a practical and cost-effective strategy for risk stratification, especially in resource-limited healthcare systems. Prospective multicenter studies are needed to validate these results externally and to explore multimodal models that also include molecular biomarkers or advanced imaging parameters.

This highlights the importance of validating simple, inexpensive prognostic markers in real-world settings, while acknowledging their limitations compared with molecular or imaging biomarkers.

## Figures and Tables

**Table 1:** Characteristics of the analyzed patients according to mortality.

Parameter	Total	Survivors	Deceased	*p*
Demographic characteristics				
N, %	304 (100%)	91 (29.9%)	213 (70.1%)	-
Gender (Male) (Nr., %)	208 (68.4%)	58 (63.7%)	150 (70.4%)	0.282 *
Age (Median (IQR))	67 (62–72)	66 (61–70)	67 (62–73)	0.056 **
Residence (Urban) (Nr., %)	182 (59.9%)	59 (64.8%)	123 (57.7%)	0.307 *
Smoking (Nr., %)				
Absent	51 (16.8%)	16 (17.6%)	35 (16.4%)	0.970 *
Smoker	158 (52%)	47 (51.6%)	111 (52.1%)	
Former	95 (31.2%)	28 (30.8%)	67 (31.5%)	
Packs-years (Median (IQR))	31 (20–45)	36 (25–50)	30 (20–40)	0.092 **
Job exposure (Nr., %)	112 (36.8%)	36 (39.6%)	76 (35.7%)	0.520 *
ECOG score (Median (IQR))	1 (0–2)	1 (0–2)	1 (0–2)	0.347 **

N—number of patients, * Fisher’s Exact Test, ** Mann–Whitney U Test.

**Table 2:** Localization of the tumor.

Localization	Total	Survivors	Deceased	*p*
Upper right lobe	74 (24.3%)	27 (29.7%)	47 (22.1%)	0.307 *
Upper left lobe	80 (26.3%)	18 (19.8%)	62 (29.1%)	
Lower right lobe	58 (19.1%)	18 (19.8%)	40 (18.8%)	
Lower left lobe	43 (14.1%)	15 (16.5%)	28 (13.1%)	
Right centrilobular	24 (7.9%)	4 (4.4%)	20 (9.4%)	
Left centrilobular	18 (5.9%)	6 (6.6%)	12 (5.6%)	
Mediastinal	7 (2.3%)	3 (3.3%)	4 (1.9%)	

* Fisher’s Exact Test.

**Table 3:** Aspect of the tumor.

Aspect	Total	Survivors	Deceased	*p*
Pulmonary node	28 (9.2%)	15 (16.5%)	13 (6.1%)	0.022 *
Pleurisy-associated tumor	80 (26.3%)	22 (24.2%)	58 (27.2%)	
Tumor mass	196 (64.5%)	54 (59.3%)	142 (66.7%)	

* Fisher’s Exact Test.

**Table 4:** Distribution of TNM stage, nodal and metastatic status.

T Staging	Total	Survivors	Deceased	*p*
T1	10 (3.3%)	3 (3.3%)	7 (3.3%)	<0.001 *
T2	69 (22.7%)	32 (35.2%)	37 (17.4%)	
T3	39 (12.8%)	17 (18.7%)	22 (10.3%)	
T4	186 (61.2%)	39 (42.9%)	147 (69%)	
N staging	Total	Survivors	Deceased	*p*
N0	72 (23.8%)	34 (37.4%)	38 (18%)	0.006 *
N1	33 (10.9%)	8 (8.8%)	25 (11.8%)	
N2	134 (44.4%)	33 (36.3%)	101 (47.9%)	
N3	63 (20.9%)	16 (17.6%)	47 (22.3%)	
M1 staging	169 (57.1%)	44 (49.4%)	125 (60.4%)	0.096 *

* Fisher’s Exact Test.

**Table 5:** Laboratory parameters (Median IQR).

Parameters	Reference Range (Laboratory Standard)	Survivors Median (IQR)	Deceased Median (IQR)	*p*
Hb	12.5 (11.4–13.7)	12.9 (12–14.2)	12.35 (11.2–13.6)	0.005 **
MCV	89 (85.1–92.9)	90.7 (86.3–94.8)	88.5 (84.7–92.1)	0.009 **
MCHC	31.5 (30.7–32.3)	31.3 (30.3–31.9)	31.6 (30.7–32.5)	0.015 **
WBC	9.98 (7.7–12.4)	8.86 (7.6–11.3)	10.3 (7.9–13.2)	0.001 **
PLT	298.5 (230–377)	276 (225–337)	308 (230–399.5)	0.021 **
Fibrinogen	4.64 (3.94–5.89)	4.49 (3.57–5.61)	4.71 (4.08–5.98)	0.032 **
ESR	50 (24–75)	33.5 (14–71)	50 (26–76)	0.185 **
CRP	12 (6–48)	6 (6–24)	12 (6–48)	0.061 **
D-dimer	1.03 (0.46–1.93)	1.16 (0.33–1.66)	1.01 (0.51–1.98)	0.285 **

Hb—Hemoglobin, MCV—mean corpuscular volume, MCHC—corpuscular hemoglobin concentration, WBC—white blood cell count, PLT—platelet count, ESR—erythrocyte sedimentation rate, CRP—C reactive protein, ** Mann-Whitney U Test.

**Table 6:** Kaplan-Meier analyses for overall survival comparison according to investigated factors.

Hb—Risk	Mean (95% C.I.)	Median (95% C.I.)	*p* *
Absent (Hb > 11.85 g/dL)	577.8 (494–661)	495 (404–585)	<0.001
Present (Hb ≤ 11.85 g/dL)	344.3 (273–415)	224 (174–273)	
MCV—Risk	Mean (95% C.I.)	Median (95% C.I.)	*p* *
Absent (MCV > 90.15 fL)	593.28 (492–694)	452 (338–565)	0.002
Present (MCV ≤ 90.15 fL)	421.8 (351–492)	279 (205–352)	
MCHC—Risk	Mean (95% C.I.)	Median (95% C.I.)	*p* *
Absent (MCHC < 31.95 g/dL)	519.3 (431–606)	379 (282–475)	0.482
Present (MCHC ≥ 31.95 g/dL)	458 (380–536)	329 (209–448)	
WBC—Risk	Mean (95% C.I.)	Median (95% C.I.)	*p* *
Absent (WBC < 11.71 × 10^9^/L)	587.9 (501–674)	470 (362–577)	<0.001
Present (WBC ≥ 11.71 × 10^9^/L)	337.3 (276–398)	243 (175–310)	
PLT—Risk	Mean (95% C.I.)	Median (95% C.I.)	*p* *
Absent (PLT < 358.5 × 10^9^/L)	568.8 (482–655)	416 (319–512)	0.001
Present (PLT ≥ 358.5 × 10^9^/L)	362 (297–427)	279 (203–354)	
Fibrinogen—Risk	Mean (95% C.I.)	Median (95% C.I.)	*p* *
Absent (Fibrinogen < 4.06 g/dL)	582.2 (458–706)	416 (258–573)	0.026
Present (Fibrinogen ≥ 4.06 g/dL)	422.3 (371–473)	329 (247–410)	

Hb—Hemoglobin, MCV—mean corpuscular volume, MCHC—corpuscular hemoglobin concentration, WBC—white blood cell count, PLT—platelet count. * Log-rank Test. Note: “Absent” indicates values below the risk threshold (within the normal or low-risk range); “Present” indicates values above the risk threshold (high-risk group).

**Table 7:** Univariable and multivariable Cox proportional hazard regression model for predicting mortality risk using investigated factors.

Parameter	Univariable		Multivariable	
	HR (95% C.I.)	*p*	HR (95% C.I.)	*p*
Tumor mass aspect	2.213 (1.261–3.885)	0.006		
T4 stage	1.708 (1.274–2.289)	<0.001	1.469 (1.078–2.002)	0.015
N1/2/3 stage	1.609 (1.131–2.287)	0.008		
Over IIC stage	2.176 (1.367–3.461)	0.001		
Metastases	1.362 (1.030–1.801)	0.030		
Hb—Risk	1.821 (1.376–2.408)	<0.001	1.702 (1.273–2.777)	<0.001
MCV—Risk	1.535 (1.160–2.030)	0.003		
WBC—Risk	1.804 (1.367–2.380)	<0.001	1.539 (1.152–2.056)	0.004
PLT—Risk	1.572 (1.186–2.084)	0.002		
Fibrinogen—Risk	1.459 (1.044–2.037)	0.027		

Hb—Hemoglobin, MCV—mean corpuscular volume, WBC—white blood cell count, PLT—platelet count.

**Table 8:** Characteristics of the analyzed patients according to the analyzed factors and formed Clusters.

Parameter (Nr., %)	Cluster 1	Cluster 2	*p* *
T4 stage	70 (40.9%)	115 (87.1%)	<0.001
HbRisk	27 (15.8%)	77 (58.3%)	<0.001
WBCRisk	20 (11.7%)	80 (80.6%)	<0.001

Hb—Hemoglobin. WBC—white blood cells. * Fisher’s Exact Test.

## Data Availability

Data supporting the findings of this study are available from the corresponding author upon reasonable request.

## Supplementary Materials

The following supporting information can be downloaded at: https://www.mdpi.com/article/10.3390/jcm14217603/s1. Table S1: Stage-specific distribution of survivors and deceased patients (TNM classification). Table S2: Kaplan-Meier analyses for overall survival. Figure S1: Comparison of hemoglobin, leukocytes, and fibrinogen levels according to mortality b. Comparison of platelet levels according to mortality. Table S3: ROC curve analyses for the prediction of mortality using laboratory parameters. Table S4: Means Cluster Analysis based on observed significant factors over mortality.

## References

[1] Sung H., Ferlay J., Siegel R.L., Laversanne M., Soerjomataram I., Jemal A., Bray F. (2021). Global Cancer Statistics 2020: GLOBOCAN estimates of incidence and mortality worldwide for 36 cancers in 185 countries. CA Cancer J. Clin..

[2] Zhou J., Xu Y., Liu J., Feng L., Yu J., Chen D. (2024). Global Burden of Lung Cancer in 2022 and Projections to 2050: Incidence and Mortality Estimates from GLOBOCAN. Cancer Epidemiol..

[3] Santucci C., Patel L., Malvezzi M., Wojtyla C., La Vecchia C., Negri E., Bertuccio P. (2022). Persisting cancer mortality gap between western and eastern Europe. Eur. J. Cancer.

[4] Peng B., Wang Y.H., Liu Y.M., Ma L.-X. (2015). Prognostic significance of the neutrophil to lymphocyte ratio in patients with non-small cell lung cancer: A systemic review and meta-analysis. Int. J. Clin. Exp. Med..

[5] Xu W., Liu X., Ci Y., Abdurahmane G., Lazibiek J., Zhang Y., Cao M. (2024). The prognostic value and model construction of inflammatory markers for patients with non-small cell lung cancer. Sci. Rep..

[6] Hong S., Kang Y.A., Cho B.C., Kim D.J. (2012). Elevated serum C-reactive protein as a prognostic marker in small cell lung cancer. Yonsei Med. J..

[7] Johansen J., Boisen M.K., Mellemgaard A. (2013). Prognostic value of ECOG performance status in lung cancer assessed by patients and physicians. J. Clin. Oncol..

[8] Ay C., Dunkler D., Pirker R., Thaler J., Quehenberger P., Wagner O., Zielinski C., Pabinger I. (2012). High D-dimer levels are associated with poor prognosis in cancer patients. Haematologica.

[9] Oken M.M., Creech R.H., Tormey D.C., Horton J., Davis T.E., McFadden E.T., Carbone P.P. (1982). Toxicity and response criteria of the Eastern Cooperative Oncology Group. Am. J. Clin. Oncol..

[10] Gomez P., Tartari C., Russo L., Bolognini S., Ticozzi C., Romeo D., Schieppati F., Barcella L., Falanga A., Marchetti M. (2025). Thromboinflammatory biomarkers are early predictors of disease progression in non-small cell lung cancer patients. Cancers.

[11] Moik F., Müller S., Posch F., Pabinger I., Ay C. (2020). Systemic inflammation and activation of haemostasis predict poor prognosis and response to chemotherapy in patients with advanced lung cancer. Cancers.

[12] Zhang Y., Wan W., Shen R., Zhang B., Wang L., Zhang H., Ren X., Cui J., Liu J. (2024). Prognostic factors and construction of nomogram prediction model of lung cancer patients using clinical and blood laboratory parameters. Onco Targets Ther..

[13] Tian H., Li G., Hou W., Jin J., Wang C., Ren P., Wang H., Wang J., Li W., Liu D. (2023). Common nutritional/inflammatory indicators are not effective tools in predicting the overall survival of patients with small cell lung cancer undergoing first-line chemotherapy. Front. Oncol..

[14] Stensvold A.M., Aggerholm-Pedersen N., Winther-Larsen A., Sandfeld-Paulsen B. (2021). Pretreatment C-Reactive Protein Predicts Survival in Small Cell Lung Cancer Patients. Onco.

[15] Zhong H., Qian Y., Fang S., Wang Y., Tang Y., Gu W. (2018). Prognostic Value of Plasma Fibrinogen in Lung Cancer Patients: A Meta-Analysis. J. Cancer.

[16] SEER Cancer of the Lung and Bronchus-Cancer Stat Facts. https://seer.cancer.gov/statfacts/html/lungb.html.

[17] Ayoade O.F., Canavan M.E., Zolfaghari E.J., Caturegli G., Kim S.Y., Boffa D.J. (2025). Recent survival gains in stage IV NSCLC by sociodemographic strata. JTO Clin. Res. Rep..

[18] Chen L., Zhao X., Wang S. (2022). Factors leading to the risk of stroke mortality: A cross-sectional study with lung cancer patient-based large sample. Eur. J. Cancer Prev..

[19] Wang C., Shao J., Song L., Ren P., Liu D., Li W. (2023). Persistent increase and improved survival of stage I lung cancer based on a large-scale real-world sample of 26,226 cases. Chin. Med. J..

[20] Li X., Tong L., Wang S., Yu J., Lu B., Wang Q., Hu M., Wu J., Yu J., Li B. (2024). Integration of clinical and blood parameters in risk prognostication for patients receiving immunochemotherapy for extensive stage small cell lung cancer: Real-world data from two centers. BMC Med..

[21] Killeen R.B., Kok S.J. (2025). D-Dimer Test.

[22] Ma M., Cao R., Wang W., Wang B., Yang Y., Huang Y., Zhao G., Ye L. (2021). The D-dimer level predicts the prognosis in patients with lung cancer: A systematic review and meta-analysis. J. Cardiothorac. Surg..

[23] Li X., Lu D., Zhang Z., Zhang Y., Wang J., Hu Y. (2022). Prognostic Value of Plasma D-Dimer Levels in Advanced Non-Small Cell Lung Cancer Patients Treated with Immune Checkpoint Inhibitors: A Retrospective Study. J. Thorac. Dis..

[24] Zhang K., Xu Y., Tan S., Wang X., Du M., Liu L. (2019). The Association between Plasma Fibrinogen Levels and Lung Cancer: A Meta-Analysis. J. Thorac. Dis..

[25] Sinn K., Mosleh B., Grusch M., Klepetko W., Hoetzenecker K., Klikovits T., Gompelmann D., Hoda M.A. (2022). Impact of fibrinogen levels and modified Glasgow pognostic score on survival of stage III/N2 non-small cell lung cancer patients treated with neoadjuvant therapy and radical resection. BMC Cancer.

[26] Mitsui S., Tanaka Y., Doi T., Hokka D., Maniwa Y. (2022). Prognostic value of preoperative plasma fibrinogen levels in resected stage I non-small cell lung cancer. Thorac Cancer.

[27] Riedl J.M., Barth D.A., Brueckl W.M., Zeitler G., Foris V., Mollnar S., Stotz M., Rossmann C.H., Terbuch A., Balic M. (2020). C-Reactive Protein (CRP) Levels in Immune Checkpoint Inhibitor Response and Progression in Advanced Non-Small Cell Lung Cancer: A Bi-Center Study. Cancers.

[28] Onodera R., Chiba S., Nihei S., Fujimura I., Akiyama M., Utsumi Y., Nagashima H., Kudo K., Maemondo M. (2023). High Level of C-Reactive Protein as a Predictive Factor for Immune-Related Adverse Events of Immune Checkpoint Inhibitors in Non-Small Cell Lung Cancer: A Retrospective Study. J. Thorac. Dis..

[29] Gong L., Zhu L., Zhang Y., Kawaguchi T., Otori T., Nakamura Y. (2023). Analysis of risk factors for febrile neutropenia in small-cell lung cancer patients receiving etoposide–cisplatin chemotherapy. Am. J. Cancer Res..

