# Supplementary material for: Prognostic Value of Routine Hematological Markers and ECOG Performance in Predicting Overall Survival in Lung Cancer—A Retrospective Cohort Study and Literature Review
Source: J Clin Med. 2025 Oct 27;14(21):7603. doi: 10.3390/jcm14217603 (PMC12608793; doi:10.3390/jcm14217603)
Supplement: Supplementary file 1 [file jcm-14-07603-s001.zip › jcm-3900446-supplementary.pdf]

3.4. TNM classification and survival

Table S1. Stage-specific distribution of survivors and deceased patients (TNM classification)

Stage	Total	Survivors	Deceased	p
IA	8 (2.6%)	2 (2.2%)	6 (2.8%)	0.005
IB	8 (2.6%)	6 (6.6%)	2 (0.9%)	
IIA	9 (3%)	6 (6.6%)	3 (1.4%)	
IIB	15 (4.9%)	7 (7.7%)	8 (3.8%)	
IIC	1 (0.3%)	0 (0%)	1 (0.5%)	
IIIA	27 (8.9%)	8 (8.8%)	19 (8.9%)	
IIIB	38 (12.5%)	11 (12.1%)	27 (12.7%)	
IIIC	28 (9.2%)	7 (7.7%)	21 (9.9%)	
IVA	100 (32.9%)	33 (36.3%)	37 (61.5%)	
IVB	47 (15.5%)	9 (9.9%)	38 (17.8%)	
IVC	23 (7.6%)	2 (2.2%)	21 (9.9%)	

Table S2. Kaplan-Meier analyses for overall survival

Tumor aspect	Mean (95% C.I.)	Median (95% C.I.)	p
Pulmonary node	745.03 (559-930)	764 (395-1132)	0.004
Tumor mass	465 (404-525)	329 (249-408)	
T4 stage	Mean (95% C.I.)	Median (95% C.I.)	p
Absent	684.17 (552-815)	473 (271-674)	<0.001
Present	401.75 (346-456)	293 (224-361)	
N1/2/3 stage	Mean (95% C.I.)	Median (95% C.I.)	p
Absent	676.44 (518-834)	495 (174-815)	0.007
Present	450.95 (389-512)	329 (249-408)	
Tumor stage	Mean (95% C.I.)	Median (95% C.I.)	p
IA/IB/IIA/IIB/IIC	794.56 (595-994)	764 (367-1160)	0.001
IIIA/IIIB/IIIC/IVA/IVB/IVC	450 (390-510)	323 (240-405)	
Metastases	Mean (95% C.I.)	Median (95% C.I.)	p

Absent	591.15 (478-703)	399 (290-507)	0.029
Present	432.25 (368-495)	32329-417)	

3.5. Hematological and inflammatory parameters in survivors vs. deceased patients

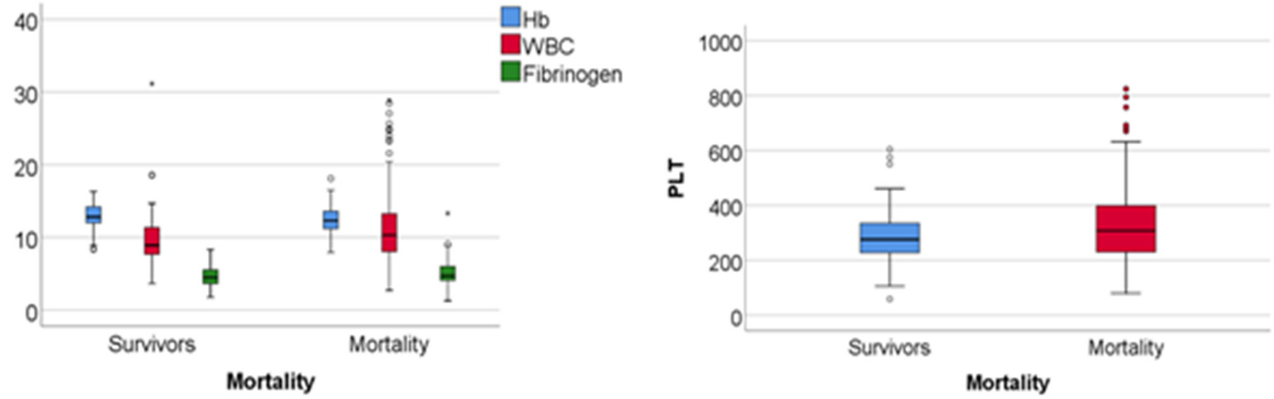

Figure S1 - Comparison of hemoglobin, leukocytes, and fibrinogen levels according to mortality Comparison of platelet levels according to mortality

Table S3. ROC curve analyses for the prediction of mortality using laboratory parameters

Parameter	AUC (95% C.I.)	Std. Error	p	Cut-off	Se	Sp
Hb	0.601 (0.531-0.671)	0.036	0.005	11.85	40.1%	79.1%
MCV	0.593 (0.522-0.664)	0.036	0.010	90.15	62.3%	56%
MCHC	0.583 (0.512-0.655)	0.037	0.025	31.95	40.1%	77%
WBC	0.615 (0.548-0.682)	0.034	0.002	11.71	41.1%	83.9%
PLT	0.595 (0.528-0.663)	0.035	0.010	358.5	38.1%	82.8%
Fibrinogen	0.580 (0.505-0.655)	0.038	0.032	4.06	77.2%	41.4%

3.7. Cluster Analysis

Table S4. Means Cluster Analysis based on observed significant factors over mortality

Parameter	Initial cluster Center 1 vs. 2	Final cluster Centers 1 vs. 2
T4 stage	0 / 1	0.41 / 0.87

HbRisk	0 / 1	0.16 / 0.58
WBCRisk	0 /1	0.12 / 0.61

Distances between Final Cluster Centers = 0.910 / 0.910
